# An Optical Sensor for Measuring In-Plane Linear and Rotational Displacement

**DOI:** 10.3390/s25133996

**Published:** 2025-06-26

**Authors:** Suhana Jamil Ahamed, Michael Aaron McGeehan, Keat Ghee Ong

**Affiliations:** 1 Department of Bioengineering, Knight Campus for Accelerating Scientific Impact, University of Oregon, Eugene, OR 97403, USAmmcgeeha@uoregon.edu (M.A.M.); 2 Department of Human Physiology, University of Oregon, Eugene, OR 97403, USA

**Keywords:** optical sensor, shear, linear displacement, rotational displacement, orthotic, prosthesis, footwear

## Abstract

We developed an optoelectronic sensor capable of quantifying in-plane rotational and linear displacements between two parallel surfaces. The sensor utilizes a photo detector to capture the intensity of red (R), green (G), blue (B), and clear (C, broad visible spectrum) light reflected from a color gradient wheel on the opposing surface. Variations in reflected R, G, B and C light intensities, caused by displacements, were used to predict linear and rotational motion via a polynomial regression algorithm. To train and validate this model, we employed a custom-built positioning stage that produced controlled displacement and rotation while recording corresponding changes in light intensity. The reliability of the predicted linear and rotational displacement results was evaluated using two different color gradient wheels: a wheel with changing color hue, and another wheel with changing color hue and saturation. Benchtop experiments demonstrated high predictive accuracy, with coefficients of determination (*R*^2^) exceeding 0.94 for the hue-only wheel and 0.92 for the hue-and-saturation wheel. These results highlight the sensor’s potential for detecting shear displacement and rotation in footwear and wearable medical devices, such as orthotics and prostheses, enabling the detection of slippage, overfitting, or underfitting. This capability is particularly relevant to clinical conditions, including diabetic neuropathy, flat feet, and limb amputations.

## 1. Introduction

There is a growing need for compact, power-efficient sensors capable of accurately measuring mechanical shear and displacement in wearable applications, particularly outside laboratory settings [1,2,3,4,5]. Previously, we developed an optoelectronic sensor system that detects the displacement between two parallel surfaces using optical signals [6,7,8]. This sensor relies on a photodetector to quantify red (R), green (G), blue (B), and clear (C) light intensities reflected from a color grid [6,7,8]. Earlier iterations of our sensor employed nine-square and four-square color grids to detect linear displacement [7,8]. While these designs could distinguish between displacements in the *x* and *y* directions, they lacked the ability to differentiate displacement directionality (e.g., +*x* vs. −*x*) or to detect rotation. McGeehan et al. [7] overcame this limitation by using a random colored grid system with a classification algorithm, while Ahamed et al. [8] improved this limitation by using a four-square grid with a linear regression model.

Compared to capacitive and resistive shear sensors [9,10,11,12], optical sensors are less susceptible to electromagnetic interference, temperature fluctuations, and humidity. Optical sensors also offer improved stability over piezoelectric sensors, which are often inconsistent in differentiating shear and compressive forces [9,10]. However, prior optical sensor designs were limited in their ability to resolve rotational displacement and were not evaluated using continuous color gradients [6,7]. This study expands upon our previous work [6,7,8] by introducing a round-shaped reflective color wheel with continuous hue and saturation gradients. The use of this gradient enhances the reliability of optical signal detection and enables the measurement of linear and rotational displacements. A polynomial regression model was implemented to map R, G, B and C intensity changes to displacement coordinates, allowing the precise measurement of linear and rotational movement. The system was evaluated under benchtop conditions using a custom positioning station. This study provides further evidence of the reliability of the prediction tool for more complex displacement conditions. Results from this paper provide further support for the applicability of this optoelectronic sensor in footwear and medical devices.

## 2. Materials and Methods

### 2.1. Sensor Component, Design and Fabrication

**Figure 1:**
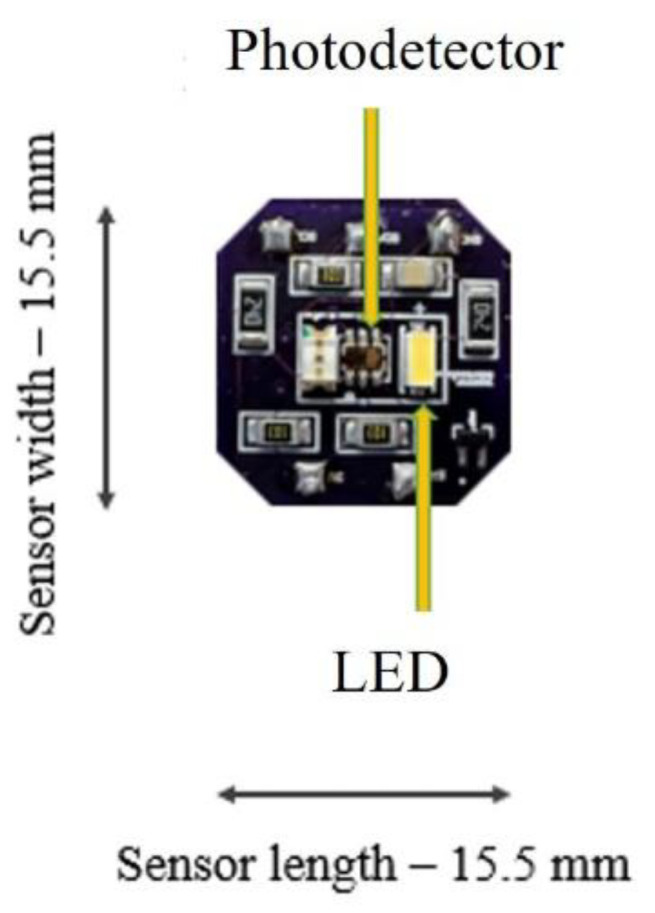
The printed circuit board (PCB) of the sensor with a photodetector and LED amongst other electronic components.

**Figure 2:**
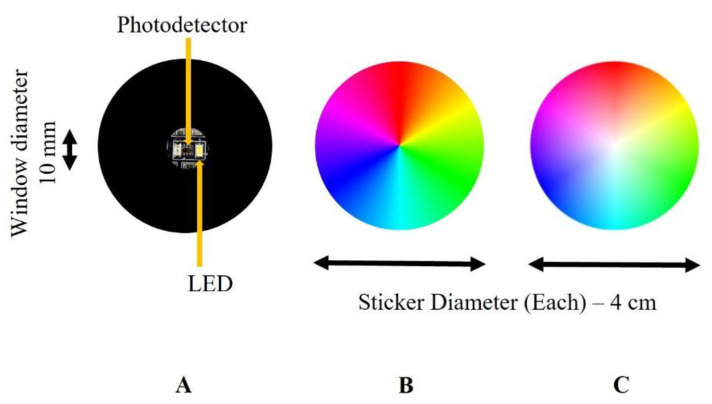
The sensor consists of a photodetector and an LED on a printed circuit board (**A**) with a round window and a color wheel with only varying hue (**B**) or a color wheel with both varying saturation and hue (**C**).

The sensor is composed of two primary components: a custom-designed printed circuit board (PCB) and a reflective color wheel (Figure 1 and Figure 2). The PCB integrates an LED (158301240, Wurth Elektronik, Yokohama, Japan) that emits visible light in the 400–800 nm spectrum, and a photodetector (TCS37727, Texas Advanced Optoelectronic Solutions, Plano, TX, USA). The LED operates at 20 mA with a luminous intensity of 2000 mcd. The photodetector includes bandpass filters to detect R, G, B, and C components, with peak responsivities at 615 nm (red), 525 nm (green), and 465 nm (blue), and a broad responsivity from 400 to 650 nm for the clear channel. Since the intensity of the LED light and the photodetector sensitivity vary with wavelengths, the measured light intensity by the photodetector would be different at the corresponding R, G, and B wavelengths. The photodetector communicates via an I^2^C interface and streams 16-bit digital data with a transmission rate up to 400 kbit/s. It provides a high-resolution measurement of light intensities, enabling robust spectral differentiation. The LED and photodetector have compact dimensions (3.0 mm × 2.0 mm × 1.4 mm and 2.0 mm × 2.4 mm × 1.6 mm, respectively) and are mounted inside Fixture A, which aligns with a circular window (10 mm diameter) as shown in Figure 1. This window allows the LED light to shine on a specific region of the color wheel and to be reflected onto the photodetector for data collection. The PCB has 4 wires: two wires for I^2^C signals consisting of a serial data line (SDA) and a serial clock line (SCL), a wire for power voltage, and a ground wire. These wires are connected to a breakout board with an Artemis module, Cortex-M4F with BLE 5.0 running up to 96 MHz, which is also connected to a laptop computer. The CNC apparatus and the PCB circuitry were operated through a Python script (IDLE PYTHON 3.7 64-bit) to control the displacement and record data with the use of the breakout board for the Artemis Module.

Two types of color wheels were fabricated and printed on matte adhesive vinyl (Jukebox Prints, Toronto, ON, Canada): one with a circular hue gradient (Figure 2B), and the other combining both hue and saturation variations (Figure 2C). A graphics editing software program (Paint.NET version 5.0.12) was used to generate and refine the gradients.

### 2.2. Operating Principle

The photodetector measures the intensity of R, G, B, and C lights when the LED light passes through the round-shaped window at a fixed optical path length and reflects from a color wheel. As the color wheel is displaced, the light intensities change due to different parts of the spectrum reflecting through the fixed optical path. The R, G, B, and C light intensities are measured when the color wheel is rotated for a full 360° cycle (120 rotational positions with an increment of 3°). Simultaneously, a linear/radial displacement is applied in increments of 1 mm, ranging from 0 mm to 10 mm, at each rotation position. This combination of rotational and linear displacements facilitates a comprehensive analysis of how light intensity changes for both angular and linear variations. Although the R, G and B spectra are equally distributed along the rotational direction, the recorded R, G and B light intensities would be most distinguishable at the linear/radial displacement of 10 mm (furthest away from the center), attributed to the larger difference in angular distance with changing rotational displacement.

**Figure 3:**
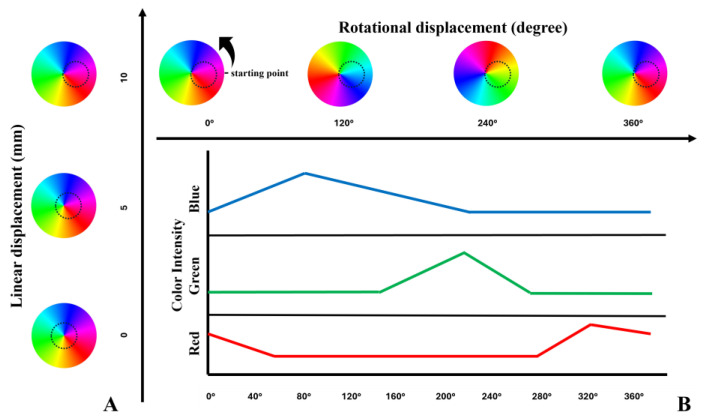
The position of the color wheel to the circular window on Fixture A (represented by the dotted circular outline) when the sensor experiences (**A**) a linear displacement and (**B**) a rotational displacement. Figure 3B illustrates the intensity of R, G and B color inside the circular window when the sensor experiences a rotational displacement from 0 to 360° (counterclockwise direction from the starting point, as indicated by the arrow next to the left-most color wheel), while the linear displacement remains at 10 mm.

Figure 3A illustrates a visible spectrum color wheel with changing hue and a dashed black circle indicating the dimension of the window at Fixture A when the PCB is linearly/radially displaced for 0 mm, 5 mm, and 10 mm from the center of the color wheel. Figure 3B illustrates the variation in the R, G and B light intensities for a full 360-degree rotational displacement cycle at a fixed linear/radial displacement of 10 mm from the center. The horizontal axis in Figure 3B represents the rotational displacement from 0° to 360°, while the vertical axis shows the light intensity for each color (R, G, B). The positions of the circular window when the color wheel is rotated at 0°, 120°, 240°, and 360° are also illustrated in Figure 3B.

As illustrated in Figure 3B, the red intensity of a hue-changing wheel decreases gradually from 0° to 55°. Because the red color remains almost absent between 55° and 280° of the color wheel, the red light intensity remains stable and low during that region. The red light intensity increases after 280°, suggesting an increase in the red hue in the color wheel in that region. In contrast, the green light intensity remains low and unchanged from the starting point to 140°. The green light intensity increases gradually to a peak of around 200° before dropping and remains relatively stable from 270° through the final phases of the rotation. The intensity of the blue color light increases gradually from the starting point to a peak around 80° and declines up to 220° and then remains stable for the remainder of the cycle, indicating the blue hue is maximum at 80° of the color wheel.

For a hue-and-saturation-changing color wheel, linear/radial displacement exposes the sensor to regions of the pattern with different saturation levels (but same hue). Rotational displacement causes the window to sweep angularly across the wheel, altering the reflected hue while retaining the same saturation level. Because the color gradients are continuous and uniquely distributed in both the radial and angular directions, any change in position results in a characteristic change in the R, G, B and C light intensity signature. By systematically mapping these spectral changes to known displacements using regression models, the system can infer the radial (linear) and angular (rotational) coordinates of the sensor relative to the color wheel. This enables the differentiation of displacement vectors in two degrees of freedom (2-DoF) without mechanical encoders or additional hardware.

### 2.3. Experimental Setup

**Figure 4:**
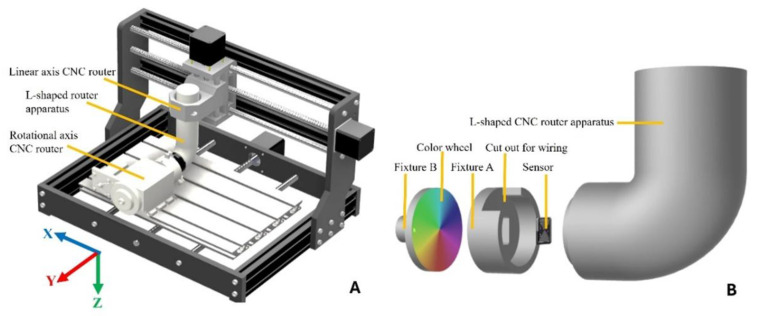
Modified CNC experiment apparatus (**A**) with disassembled components (**B**).

The sensor measures the variation in R, G, B, and C light in response to applied linear/radial or rotational displacements through a 4-axis positioning station comprising two computed numeric control (CNC) routers. Specifically, a CNC router (FoxAlien, 3018-SE V2, Moreno Valley, CA, USA) was used to provide a linear/radial displacement through an L-shape apparatus, and another CNC router (CNCTOPBOAS, Los Angeles, CA, USA) was attached to the color wheel at Fixture B to provide an anticlockwise rotational displacement, as shown in Figure 4A,B. The CNC apparatus was operated through a Python script to provide displacement and record data.

### 2.4. Data Collection and Preprocessing

To demonstrate the reliability and consistency of the sensor measurements, two data sets were collected: one using the color wheel with varying hue, and the other using the color wheel with both varying saturation and hue. The linear/radial and rotational displacement resolutions were selected to demonstrate the sensor’s precision and reliability for real-world applications. For each linear position at a displacement range of 0 to 10 mm with 1 mm resolution, 11 data points were streamed at 121 rotational positions with a rotational displacement of 3° to complete a full cycle. The recording at these 121 positions was repeated three times, resulting in 3993 data points for each light color and displacement position for both color wheel data sets. No significant variability or inconsistencies were observed within the data throughout ten consecutive measurements, confirming the reliability and stability of the experimental setup before proceeding with further analyses. To observe the best performance of the sensor, all data was collected under no ambient light, which is consistent with the intended application of this technology as an embedded sensor within shoe insole layers. To examine the effect of a non-parallel or misaligned sensor and color wheel, the PCB layer was tilted at a 5° angle from the color grid layer, followed by measuring the intensity of the R, G, B, and C light spectra. It was found that the changes in light intensity were less than 5% for a small tilt angle of 5°. We also verified the rotational consistency by setting a reference point at 0° and verifying that the color wheel returned to the reference point at every rotation.

**Figure 5:**
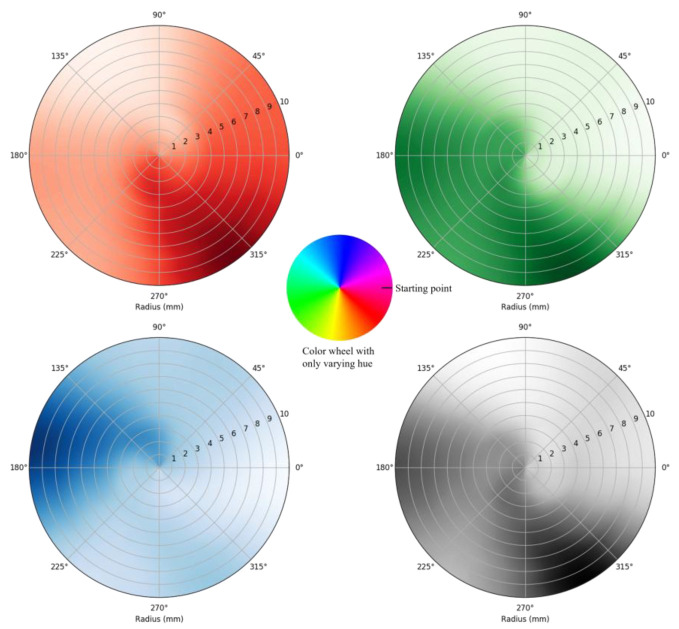
Measured red, green, blue, and clear color light intensity for a complete cycle of 360° rotational displacement and 0 to 10 mm linear displacement range as a function of in-plane displacement between the PCB surface and color wheel, with only varying hue.

**Figure 6:**
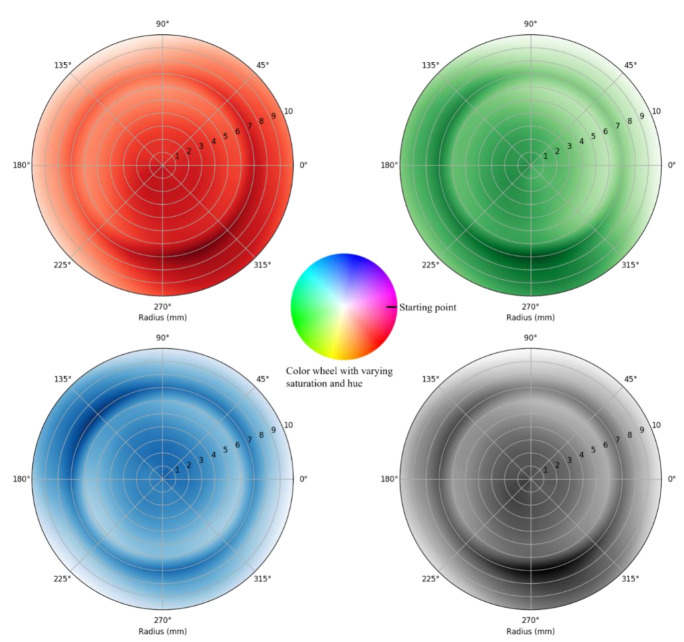
Measured red, green, blue, and clear color light intensity for a complete cycle of 360° rotational displacement and 0 to 10 mm linear displacement range as a function of in-plane displacement between the PCB surface and color wheel with varying saturation and hue.

The average of three data points is calculated for each position for the two data sets. Figure 5 and Figure 6 plot the R, G, B, and C color intensity data of a color wheel with varying hue and the data of a color wheel with varying saturation and hue, respectively. The intensity of each color is measured as a unitless 16-bit number, where 0 represents no light and 65,535 is the brightest measurement before saturation. Overall, light intensities of R, G, B and C spectra correspond with the hue and/or saturation of color at the wheels as shown in Figure 2.

Specifically, Figure 5 demonstrates a decrease in red color intensity as the rotational displacement occurs from the starting point in an anticlockwise direction and almost diminishes towards 90°. Note that the recorded red color intensity is higher towards the farthest radial displacement at about 315°, correlating to the higher red hue on the color wheel used in the experiment (Figure 5, at the center). Meanwhile, there is an increase in blue color intensity with continuing rotational displacement through the intermediary hue from the starting point (Figure 5). The blue color light intensity increases through the transition from magenta color to blue color from about 30° to 180° (Figure 5). In addition, the gradual decrease in the blue color from 180° to 225° occurs as the blue color transitions into turquoise color (Figure 5). This gradual change is more significantly visible with the linear/radial displacement of 10 mm (i.e., outermost circumference of the graph).

However, the green color light intensity follows a different trajectory than the illustrated theoretical framework (Figure 5). The green color intensity shows no change from 135° to 225°, indicating that blue and turquoise have some component of green color in them. The intensity of the green color appears higher between 270° and 315°, relating to the color wheel with varying saturation (Figure 5). This data deviation resonates with the photodetector’s responsivity, which is 80–110% (ratio of minimum/maximum responsivity to clear channel value) at 615 nm for the red color, 60–85% at 525 nm for the green color, and 65–88% at 465 nm for the blue color, respectively. The clear channel detects light across a broad spectrum, with balanced responsivity between 11 and 23.4 counts/μW/cm^2^. The percentage range represents the photodetector’s efficiency or sensitivity variation at each wavelength. A higher responsivity range suggests a stronger detection capability at that wavelength, meaning the photodetector can detect lower light levels more accurately at that specific color.

Similarly, the data collected from the color wheel with varying saturation and hue exhibits a trend of continuously increasing intensity from the center toward the periphery (Figure 6). Although it is difficult to interpret the transit amongst the hues in both linear and rotational directions, the peak responsivity for each color is like the data set of a color wheel with varying saturation. In Figure 5 and Figure 6, some abrupt transitions and deviations from the illustrated trajectories of red, green, blue, and light color intensity, are observed at multiple coordinates. This may be due to the LED’s asymmetric positioning, the mismatches between the color on the grid surface, and the light wavelengths of the LED for its corresponding color. There is also a possibility of a mismatch of color ratio used in printing the color wheel and sensing color elements, which are salient with varying hue and saturation. It is interesting to note that clear light intensity shows uniform distribution only with the data set of the color wheel with varying saturation and hue (Figure 6). This could be because of the clear light intensity being predominantly present throughout the continuous color wheel. Typically, clear light intensity detected between a balanced range between 11 and 23.4 counts/μW/cm^2^ suggests a stable performance and a baseline level of responsivity that can be referenced for consistency or for detecting shifts in overall light intensity. Upon further observation, it was observed that clear light intensity shows a dense distribution like red color light intensity distribution in both data sets. However, these patterns are neither linear nor distinct. Thus, a fitting algorithm was used on the R, G, and B color intensities as inputs to generate rotational and linear displacement coordinates as outputs (radial and theta). The following section focuses on the algorithm used for this study, i.e., the polynomial regression model in machine learning.

#### 2.4.1. Polynomial Regression Model

**Figure 7:**
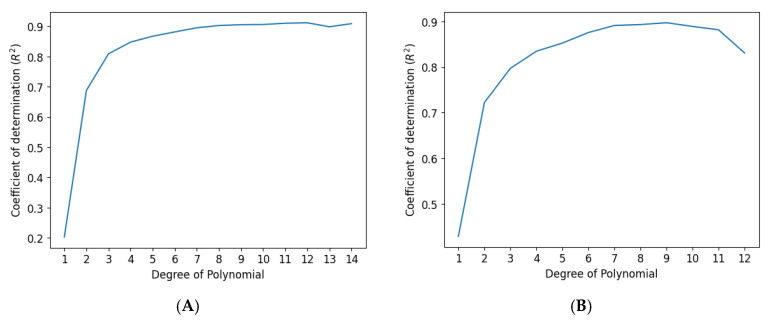
Graphs present *R*^2^ at different degrees of order for data sets from the color wheel with varying hue (**A**) and the color wheel with varying saturation and hue (**B**). The graphs demonstrate the highest *R*^2^ value of 0.94 at the polynomial of degree 12 for the color wheel with varying hue (**A**) and the highest *R*^2^ value of 0.92 at the polynomial of degree 9 for the color wheel with varying saturation and hue (**B**).

In previous studies, only linear displacement data from the CNC device and sensor outputs (R, G, B, and C intensities) were used to design prediction models, in which displacements occurred in the horizontal, vertical, or diagonal direction [12]. In this study, the same sensor outputs, R, G, B and C color intensity, are used to predict absolute rotational (radial) and linear coordinate displacement, determining the magnitude and direction of shear displacement. Both data sets capture non-linear patterns. The polynomial regression introduces higher-order terms (e.g., quadratic, cubic) used in models for such non-linear complex data sets. However, overfitting can be a concern with higher orders, so careful model design is necessary. Therefore, the data set is split into training, validation, and test sets, with the majority reserved for training to analyze relationships. The validation set is used to fine-tune parameters, and the test set evaluates the model’s performance. Key metrics for assessing the model include the coefficient of determination (*R*^2^). Figure 7A,B illustrate the model’s performance with varying polynomial degrees for data sets from discrete and continuous color wheel gradients.

#### 2.4.2. Model Fitting

For this study, different data split ratios and randomized values were tested to avoid algorithm bias using Python programming and sci-kit (SK) learn libraries. In addition, the algorithm’s predictive performance was also assessed on different degree orders with different split ratios and randomized values (random_state variable syntax used in Python). Different split ratio combinations were tested for the random_state value between 30 and 90, and polynomial degree orders between 1 and 20. This paper presents the algorithm’s results with 70%, 15%, and 15% data split ratios, among the training, test, and validation sets, with a random_state value of 35 and 46 for the two data sets: one with color wheel varying hue and another with color wheel varying saturation and hue, respectively. These split ratios and random_state values resulted in the most optimized performance without algorithm bias. Two separate polynomial models were designed for the data sets from the two-color wheels. Figure 7A,B plot the performance assessment of the models using *R*^2^ at different degrees of order for the data sets from the color wheel with varying hue and the color wheel with varying saturation and hue, respectively. The highest *R*^2^ value for the data from the color wheel with varying hue occurs at the degrees of polynomials 12 in Figure 7A. The highest *R*^2^ value for the data from the color wheel with varying saturation and hue occurs at the degrees of polynomials 9 in Figure 7B.

## 3. Results

### 3.1. Light Intensity Trends

As shown in Figure 5 and Figure 6, the R, G, B, and C channel responses exhibited displacement-dependent changes that were consistent across color wheels. The red light intensity decreased from 90° to 150°, plateaued across mid-range angles, and then increased again beyond 280°. The blue light intensity peaked around 80°, while the green light intensity showed a broad maximum near 200°, with relatively stable readings beyond 270°. The clear channel demonstrated less pronounced variation but still tracked consistent changes in combined RGB reflectivity.

Intensity gradients were more evenly distributed across the hue-and-saturation wheel compared to the hue-only wheel, suggesting that saturation variation improved spectral sensitivity. This observation aligns with the improved stability observed in the corresponding regression model.

### 3.2. Prediction Accuracy

**Figure 8:**
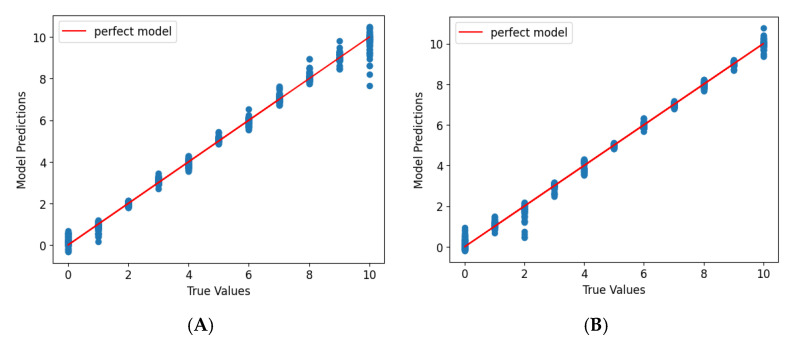
Graphs show the model prediction and true values of linear displacement coordinates for data from discrete gradient color wheel (**A**) and continuous gradient color wheel (**B**) at polynomials of degrees 12 and 9, respectively.

**Figure 9:**
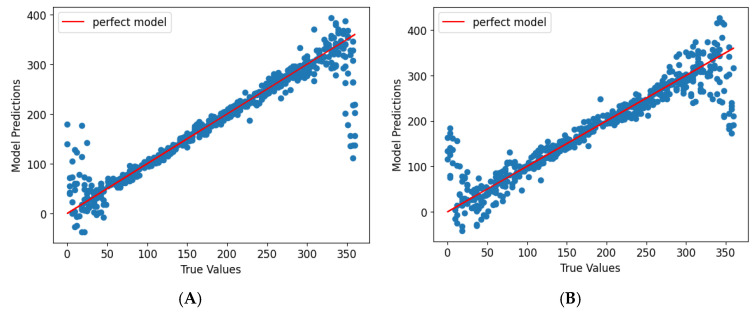
Graphs show the model prediction and true values of rotational displacement coordinates for data from discrete gradient color wheel (**A**) and continuous gradient color wheel (**B**) at polynomial of degrees 12 and 9, respectively.

The prediction performance was assessed using scatter plots comparing model predictions to actual displacement coordinates (Figure 8A,B for linear, Figure 9A,B for rotational displacement). Both models demonstrated strong linearity with only minor deviations, particularly near color transitions. The hue-only wheel exhibited slightly lower accuracy in rotational displacement prediction, especially near ±90°, where hue gradients transitioned rapidly and RGB overlap occurred.

The model accuracy improved with the inclusion of saturation in the second wheel design. Rotational displacement predictions using the hue-and-saturation wheel were more uniformly distributed across the full 360° range, suggesting that the added color dimensionality enhanced prediction stability.

### 3.3. Outlier and Responsivity Considerations

Prediction outliers were generally localized to regions of high color overlap or non-linear hue transitions. These errors were most prevalent near angular sectors where red and blue values intersected, such as around 60° and 120°. The sensor’s internal responsivity also played a role in signal variation. According to the manufacturer datasheet, the photodetector’s responsivity varies from 65 to 88% at 465 nm (blue), 60 to 85% at 525 nm (green), and 80 to 110% at 615 nm (red). The clear channel offers a flatter response profile, with output ranging from 11 to 23.4 counts/µW/cm^2^ across the 400–650 nm range.

Although the variation in responsivity introduced some non-linearity into signal measurements, these effects were largely corrected through polynomial regression. Additional improvements in print calibration, LED emission uniformity, and angular alignment are expected to reduce outlier frequency in future iterations.

## 4. Discussion

This study demonstrates the feasibility of a compact, low-cost optoelectronic sensor to simultaneously detect linear and rotational displacement using spectral reflectance from a patterned color wheel. Compared to prior implementations of grid-based optical sensors [6,7,8], the use of continuous color gradients offers improved resolution, directionality, and robustness. The sensor’s response to displacement was consistent and highly predictable, as evidenced by high *R*^2^ values obtained from polynomial regression models trained on R, G, B and C intensity data.

A key advancement of this work is the sensor’s ability to resolve rotational and linear displacements when the rotational axis is at the center of the color wheel. By leveraging the unique mapping between position on the color wheel and spectral reflectance, the sensor infers displacement with sub-degree angular and sub-millimeter linear precision. This approach eliminates the need for external encoders or mechanical linkages and reduces the overall size and power requirements of the system.

Misalignment tests confirmed the sensor’s tolerance to modest angular deviations, making it suitable for integration in real-world wearable environments where perfect alignment cannot always be guaranteed. The low variation in output (<5%) during tilt testing supports the system’s mechanical robustness.

Despite strong model performance, outliers were observed in regions of rapid hue transition or overlapping RGB values. These prediction errors were most common near ±90°, where multiple color channels contributed overlapping signals. The observed variation can be partly attributed to the photodetector’s wavelength-dependent responsivity and the printer’s limited fidelity in reproducing saturated colors. Future iterations may benefit from more precise gradient fabrication using higher-resolution printing or inkjet-based deposition to control optical density.

While the current test setup has shown the sensor responses from displacements between the sensor layers, it did not quantify the sensor responses from shear force. In our following work, a different test setup will be constructed to evaluate sensor response with respect to the actual shear force. Specifically, a mechanical tester will be used to apply shear forces that generate linear/rotational displacements between the sensor layers. Instead of displacement data, the machine learning algorithm will train with shear force as inputs.

Additional enhancements could include onboard microcontroller integration for real-time regression processing and wireless communication modules (e.g., Bluetooth Low Energy) for streaming displacement data to mobile devices. These features are critical for wearable biomedical applications such as pressure-sensing insoles, prosthetic limb alignment monitors, and rehabilitation assessment tools.

## 5. Conclusions

We developed and validated an optoelectronic displacement sensor capable of detecting in-plane linear and rotational motion between parallel surfaces. The system utilizes reflected spectral light from a circular color gradient wheel and achieves high prediction accuracy using a polynomial regression model trained on R, G, B and C intensities. Across two color wheel configurations, the sensor achieved *R*^2^ values of 0.94 and 0.91, confirming the viability of this approach.

Key benefits of the system include its ability to be miniaturized, immunity to electromagnetic interference, high spectral resolution, and tolerance to installation misalignment. These characteristics make the sensor well-suited for integration into wearable systems where traditional displacement sensors are infeasible. Future work will explore multichannel sensor arrays for distributed sensing, extended displacement ranges, and closed-loop feedback applications. The miniaturization of the sensor may also be achieved by incorporating fiber optic cable interfaces on the LED and photodetector to focus and direct the light beam, which will lead to a reduction in the window size and color wheel dimension. Overall, this platform represents a promising solution for non-invasive, real-time monitoring of displacement in biomedical and human–machine interface applications. For example, the real-time monitoring of shear forces through orthotic footwear and prosthetics would be useful in preventing clinical complications among individuals with amputated limbs, diabetic feet, and flat feet [13,14,15], while also helping prevent repetitive strain injuries and improve ergonomic practices amongst sports professionals [16,17] and aging-related concerns [18,19,20].

## Data Availability

Data will be made available upon reasonable request to the authors.
